# An unwanted intruder

**DOI:** 10.1007/s12471-020-01428-8

**Published:** 2020-05-11

**Authors:** F. F. Gonçalves, L. F. Seca, J. I. Moreira

**Affiliations:** Hospital of Vila Real, Cardiology Department, Centro Hospitalar de Trás-os-Montes e Alto Douro, Vila Real, Portugal

## Answer

**Fig. 1:**
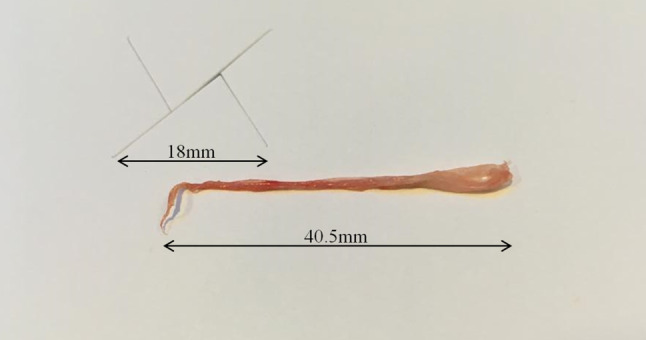
Endothelial tissue manually aspired from the left obtuse marginal artery

While suspecting a thromboembolic event and in order to restore adequate coronary flow, the operator decided to urgently perform manual aspiration, which retrieved an approximately 40-mm structure compatible with radial artery endothelial tissue (Fig. 1). After pre-dilatation with a 2.5 × 15-mm semi-compliant balloon, a 3.5 × 23-mm drug-eluting stent was implanted, with no residual stenosis and TIMI grade flow 3. Radial artery pulse was absent after sheath removal, but distal perfusion to the hand was not compromised.

Radial artery avulsion is a rare complication of transradial access in coronary angiography [1]. Moreover, embolisation of radial endothelial tissue to a coronary artery is an even more unlikely event. Radial avulsion is usually related to sheath withdrawal or spasm with guiding catheter entrapment and vessel trauma caused by its tip [2]. This can be prevented by decreasing artery spasm with careful selection and crossing of guiding catheters, as with intra-arterial administration of vasodilators. Artery avulsion is also more common in small-calibre and tortuous arteries [3]. Pre-procedural Barbeau or Allen tests are advisable in order to prevent more serious complications like hand ischaemia.

## Caption Electronic Supplementary Material

Coronary angiogram after stent implantation

## References

[1] Chugh SK, Chugh Y, Chugh S (2015). How to tackle complications in radial procedures: tip and tricks. Indian Heart J.

[2] Dandekar VK, Vidovich MI, Shroff AR (2012). Complications of transradial catheterization. Cardiovasc Revasc Med.

[3] Allam VK, Allam AK, Sameeraja V, Pramod MK (2017). Avulsion of radial artery during coronary angiogram—a case report. IHJ Cardiovasc Case Rep.

